# A New Zebrafish Model for Pseudoxanthoma Elasticum

**DOI:** 10.3389/fcell.2021.628699

**Published:** 2021-03-09

**Authors:** Dávid Czimer, Klaudia Porok, Dániel Csete, Zsolt Gyüre, Viktória Lavró, Krisztina Fülöp, Zelin Chen, Hella Gyergyák, Gábor E. Tusnády, Shawn M. Burgess, Attila Mócsai, András Váradi, Máté Varga

**Affiliations:** ^1^Department of Genetics, ELTE Eötvös Loránd University, Budapest, Hungary; ^2^Department of Physiology, Faculty of Medicine, Semmelweis University, Budapest, Hungary; ^3^Division of Biosciences, University College London, London, United Kingdom; ^4^Institute of Enzymology, Research Centre for Natural Sciences, Budapest, Hungary; ^5^Key Laboratory of Tropical Marine Bio-resources and Ecology, South China Sea Institute of Oceanology, Chinese Academy of Sciences, Guangzhou, China; ^6^Translational and Functional Genomics Branch, National Human Genome Research Institute, Bethesda, MD, United States

**Keywords:** zebrafish, PXE model, *ABCC6* gene, calcification, disease model

## Abstract

Calcification of various tissues is a significant health issue associated with aging, cancer and autoimmune diseases. There are both environmental and genetic factors behind this phenomenon and understanding them is essential for the development of efficient therapeutic approaches. Pseudoxanthoma elasticum (PXE) is a rare genetic disease, a prototype for calcification disorders, resulting from the dysfunction of ABCC6, a transport protein found in the membranes of cells. It is identified by excess calcification in a variety of tissues (e.g., eyes, skin, arteries) and currently it has no cure, known treatments target the symptoms only. Preclinical studies of PXE have been successful in mice, proving the usefulness of animal models for the study of the disease. Here, we present a new zebrafish (*Danio rerio*) model for PXE. By resolving some ambiguous assemblies in the zebrafish genome, we show that there are two functional and one non-functional paralogs for *ABCC6* in zebrafish (*abcc6a, abcc6b.1*, and *abcc6b.2*, respectively). We created single and double mutants for the functional paralogs and characterized their calcification defects with a combination of techniques. Zebrafish deficient in *abcc6a* show defects in their vertebral calcification and also display ectopic calcification foci in their soft tissues. Our results also suggest that the impairment of *abcc6b.1* does not affect this biological process.

## Introduction

Pseudoxanthoma elasticum (PXE) is a rare, autosomal recessive disorder (OMIM 264800) linked to mutations occurring in the *ATP-Binding Cassette sub-family C member 6* (*ABCC6*) gene (Bergen et al., 2000; Le Saux et al., 2000; Ringpfeil et al., 2000). PXE patients show ectopic mineralization in their connective tissues, calcification in the eyes and arteries and often develop skin lesions (Li et al., 2009; Uitto et al., 2010).

The *ABCC6* gene encodes a transmembrane efflux transporter which is expressed in the liver, kidneys, and to lesser extent is also present in blood cells (Scheffer et al., 2002; Beck et al., 2005). ABCC6 has three transmembrane domains (TMD0, TMD1, and TMD2) and two catalytic nucleotide binding domains (NBDs). TMD0 most likely has a regulatory role in the function of the transporter, whereas TMD1 and TMD2 are part of the core transporter. The NBD domains are similar to those of other ATP-binding cassette transporters (ABCs), where their role is to energize substrate transport via ATP hydrolysis. Both NBDs of ABCC6 contain highly conserved Walker A and B motifs and also a unique signature motif, both essential for their activity (Walker et al., 1982; Geourjon et al., 2001).

While the substrate of ABCC6 has not yet been confirmed, multiple lines of evidence support that it has a primary role in mammals in facilitating ATP-release from hepatocytes. ATP (and other nucleotides) is promptly cleaved in the liver vasculature to AMP and pyrophosphate by the ectonuclease *Ectonucleotide Pyrophosphatase/Phosphodiesterase 1* (*ENPP1*) (Jansen et al., 2014). Pyrophosphate has an essential role in controlling ectopic mineralization. Also supportive of ABCC6’s role in preventing calcification is that in some cases ABCC6 mutations result in a more severe set of clinical symptoms known as generalized arterial calcification of infancy 2 (GACI2, OMIM 614473) (Nitschke et al., 2012). GACI2 is phenotypically highly similar to GACI1 (OMIM 208000) which is caused by mutations in the *ENPP1* gene (Rutsch et al., 2003) and both result in extensive ectopic vascular calcification, often fatal neonatally. Animal models have confirmed an essential role of both ABCC6 and ENPP1 in the regulation of biomineralization, through the ATP – PPi pathway (Dedinszki et al., 2017; Borst et al., 2019).

Development of the *Abcc6*^–/–^ mouse models (Gorgels et al., 2005; Klement et al., 2005) resulted in fundamental discoveries related to the pathomechanism of PXE. In addition a number of attempts have been made to find or create suitable zebrafish models (Li et al., 2010; Mackay et al., 2015; Van Gils et al., 2018; Sun et al., 2021). Thanks to recent methodological developments and a number of advantageous features, the popularity of zebrafish as a pre-clinical disease model has been increasing over the past two decades (Lieschke and Currie, 2007; Varga et al., 2018). Its small size and fecundity also makes it an ideal organism to perform screens of small molecular compounds, which can facilitate drug discovery (Peterson et al., 2004; Zon and Peterson, 2005; Baraban et al., 2013). Furthermore, recent advances in micro-computer tomography (micro-CT) have made in-depth phenomic analysis of the zebrafish skeletal system possible (Grimes et al., 2016; Charles et al., 2017; Hur et al., 2017). All these features make zebrafish an ideally suited organism to model diseases that cause ectopic mineralization, such as PXE and test for possible cures.

The zebrafish genome contains two functional ABCC6 paralogs (see below), Abcc6a (65% similar and 48% identical to ABCC6) and Abcc6b.1 (63% similar and 47% identical to its human counterpart). The presence of the two paralogs could be due to a teleost-specific whole genome duplication event (TGD) that occurred approximately 320 million years ago (Mya). The TGD was followed by a rapid reshaping of the genome and differential gene loss (Glasauer and Neuhauss, 2014; Inoue et al., 2015). This historical sequence of events could explain why ∼20% of human genes have two paralogs in the zebrafish genome. These paralogs can undergo subfunctionalization, neofunctionalization or dosage selection (Glasauer and Neuhauss, 2014), something that needs to be clarified when studying such paralogous gene pairs.

Over the years a number of different models for PXE and GACI have been developed and/or identified in zebrafish. First anti-sense morpholino oligonucleotides have been used to knock down the function of endogenous *abcc6a* and *abcc6b*, and human *ABCC6* mRNA variants were injected to test their rescuing effect (Li et al., 2010). Later spontaneous loss-of-function mutations have been identified both in *abcc6a* (*gräte, grt*) and *enpp1* (*dragonfish, dgf*), and the ectopic mineralization both in *grt* and *dgf* homozygous mutants has been extensively characterized (Apschner et al., 2014; Mackay et al., 2015). The latter two studies are also notable as they have used the mutants to screen for potential rescuing effects of vitamin K and the pyrophosphate analog etidronate, respectively. Finally, CRISPR/Cas9 and TALEN induced *abcc6a* mutant alleles have been also characterized more recently (Van Gils et al., 2018; Sun et al., 2021).

However, all these earlier zebrafish approaches have their limitations. Multiple lines of evidence support that morpholino approaches are prone to off-target effects (Schulte-Merker and Stainier, 2014; Kok et al., 2015; Stainier et al., 2017), and due to their high homology, it remained as an open question if the compensating effect of the *abcc6b* for *abcc6a* loss-of-function mutations modified the phenotype (to some extent). Therefore we decided to create mutants where the function of all *ABCC6* paralogs have been abrogated.

## Results

### Evolutionary Origins of Zebrafish ABCC6 Paralogs

**FIGURE 1:**
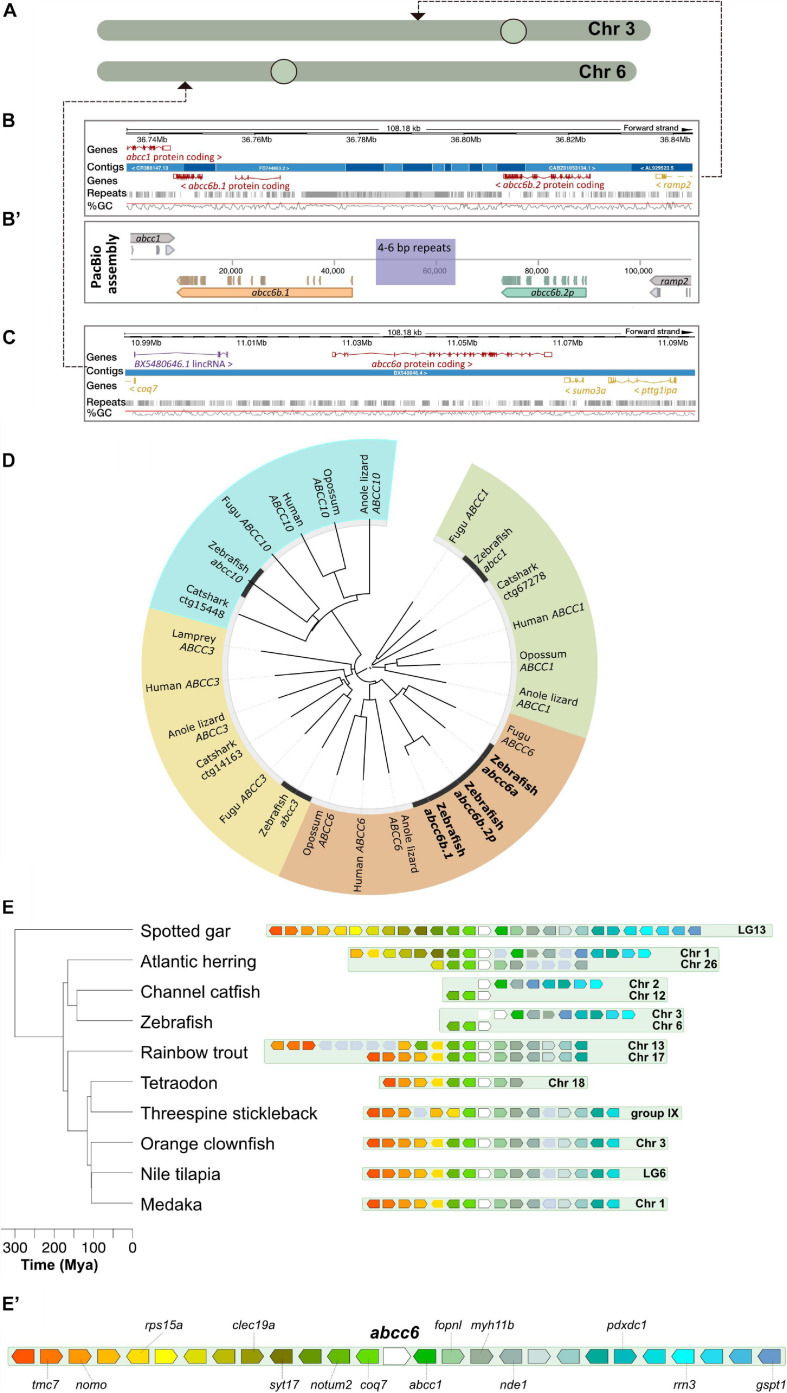
Annotation and phylogenetic relationships of the zebrafish *ABCC6* orthologs. **(A)** Chromosomal positions of zebrafish *ABCC6* orthologs. **(B,B’)** Ensembl (GRCz11) assembly of the respective chromosomal 3 regions with *abcc6b.1* and *abcc6b.2p* and reassembly of this genomic region using PacBio reads. Note the abundance of repeats in the interval between abcc6b.1 and abcc6b.2p coinciding with the highly fragmented Ensembl assembly. **(C)** Ensembl (GRCz11) assembly of the *abcc6a* containing chromosomal six region. **(D)** Phylogenetic analysis of zebrafish *ABCC1, ABCC3, ABCC6*, and *ABCC10* paralogs. **(E,E’)** Syntenic analysis of the *abcc6* genomic region in different teleost species using the spotted gar genome as reference (see text for details).

In the current version of the zebrafish genome assembly (build GRCz11) multiple potential *ABCC6* orthologs are annotated: *abcc6a* on chromosome 6, and *abcc6b.1* and *abcc6b.2* on chromosome 3 (Figures 1A–C). In order to create a novel (and potentially more revealing) zebrafish model of PXE we decided to examine if all three genes are functional and create a mutant line where every functional paralog is disabled.

Whereas previous studies have already demonstrated that *abcc6a* is functional and it’s loss-of-function can lead to adverse effects in skeletal development (Mackay et al., 2015; Van Gils et al., 2018) the functionality of only one of the potential *abcc6b* paralogs has been previously addressed and only during early development by morpholino knock-down experiments (Li et al., 2010). These two putative paralogs can be found in a repeat-rich area of chromosome 6 and previous attempts to determine whether they are functional orthologs have been hampered by the poor quality of the genome assembly in this region (Figure 1B). The fusion of multiple short contigs in this region even raised the possibility that instead of two paralogs only one is present on chromosome 6.

First we re-assembled the relevant chromosomal interval using PacBio long reads from genomic DNA of an adult “double heterozygous” heat-shock diploid zebrafish generated from the NHGRI-1 line (LaFave et al., 2014). Our results show that indeed two *ABCC6* paralogs, separated by a ∼10 kilobase long repetitive region can be identified on the reverse strand of chromosome 6 (Figure 1B’). However, as the assembled open reading frame (ORF) sequences show, of the two paralogs only *abcc6b.1* is functional, whereas the other copy is a pseudogene, hereafter called *abcc6b.2p*.

Phylogenetic analysis suggests that *abcc6b.1* and *abcc6b.2p* are the result of a recent segmental gene duplication whereas their ancestral gene and *abcc6a* arose earlier as duplicates of the original, single vertebrate *ABCC6* ortholog (Figure 1D; Parreira et al., 2018).

To reconstruct the evolution of the genomic loci on chromosomes 3 and 6 that contain *abcc6a* and *abcc6b.1*, respectively, we used the online Genomicus database (Louis et al., 2013). We used spotted gar (*Lepisosteus oculatus*) as reference as previous analyses showed that this species is a suitable outgroup to study the origin and evolution of *ABCC6* in vertebrates (Parreira et al., 2018).

The spotted gar lineage diverged from the teleosts prior to TGD (Braasch et al., 2016). Yet, our analysis shows that most extant teleost genomes contain only one genomic region syntenic to the spotted gar LG13 containing the gar *abcc6* (Figures 1E,E’). Both in the orange clownfish (*Amphiprion percula*) and threespine stickleback (*Gasterosteus aculeatus*) draft genomes (Nemo_v1 and BROAD S1, respectively) an additional potential paralog for the gene has been annotated to poorly assembled, non-syntenic telomeric genomic regions on Chromosome 16 and Group XX, respectively (Jones et al., 2012; Lehmann et al., 2019). While these might be the result of independent gene duplication events, they might also be artifacts.

Interestingly, even Salmonids such as the rainbow trout (*Oncorhynchus mykiss*), where the lineage underwent a fourth round of genome duplication (Berthelot et al., 2014) contain only two syntenic regions, suggesting that shortly after the TGD one half-genome containing the *abcc6* locus was lost in the teleost ancestor.

Of note, on zebrafish chromosome 6 only genes that are upstream of the gar *abbc6* can be identified in syntenic positions near *abcc6a*, whereas on chromosome 3 in the vicinity of *abcc6b.1* and *abcc6b.2p* only the orthologs of genes downstream of the gar *abcc6* can be found (Figures 1E,E’). A similar pattern can be observed in the related channel catfish (*Ictalurus punctatus*) as well and such an arrangement could be parsimonious with a segmental duplication of *abcc6* occurring in the ancestral zebrafish lineage followed by a chromosomal translocation breaking the cluster between the two paralogs.

However, we also noted the presence of two syntenic clusters in the Atlantic herring (*Clupea harengus*) genome. As there are no signs for an additional genome duplication event in this lineage (Pettersson et al., 2019), the presence of the two clusters might suggest either a lineage-specific chromosomal duplication event, or an independent evolution after the TGD of the Otocephala and Euteleostei clades (see Discussion for details).

In summary our analysis suggests that currently available genomic data is ambiguous about the origin of zebrafish *ABCC6* paralogs. They could have arose independently of the TGD, as the result of repeated segmental duplications, but there could have been also differential gene-loss in different ancestral Teleost lineages post-TGD, with the ancestors of zebrafish initially maintaining two syntenic *abcc6* clusters which underwent later asymmetric, complementary deletions.

### No Compensation Between Abcc6a and Abcc6b.1

In order to study the effects of complete impairment of Abcc6 function on zebrafish development we decided to create double mutants for the functional paralogs. Using CRISPR/Cas9-mediated genome editing we created frameshift alleles for both genes. In *abcc6a* we successfully targeted the second exon creating a 5 bp deletion c.175_179delGCCGA (*elu15*), that results in the p.Arg60Serfs^∗^183 mutation which disrupts the TMD0 and creates a premature termination codon (PTC). Successful editing of the fifth exon of *abcc6b.1* resulted in the c.616_618delTGTinsCTAGCAC mutation (*elu16*), creating the frame-shift p.Cys205Leufs^∗^4 in the CDS, also resulting in a PTC right after the TMD0 (Supplementary Figure 1).

Recent experiments suggest that the presence of PTCs can induce an upregulation of existing paralogs. This phenomenon, called transcriptional adaptation, might be mediated by small RNAs that appear as a result of the non-sense mediated decay (NMD) triggered by the PTCs (Rossi et al., 2015; El-Brolosy and Stainier, 2017; El-Brolosy et al., 2019; Sztal and Stainier, 2020). In order to understand if the *elu15* and *elu16* alleles could trigger similar effects, we tested if NMD could be observed in the mutants, and if the other paralog was upregulated in them.

Our results did not show signs of NMD occurring in *abcc6a* embryos or larvae, nor a compensatory upregulation of *abcc6a* in *abcc6b.1^–/–^* animals (Supplementary Figure 1C). (Due to high sequence homology, we are not able to test accurately the expression of *abcc6b.1* independently of *abcc6b.2p*, which is also expressed at these stages.)

### Larval Phenotypes

Previous studies have already provided evidence about the ectopic mineralization defects observable in *abcc6a* mutant and morphant animals (Mackay et al., 2015; Van Gils et al., 2018). First, we tested if we could see similar effects in our *abcc6a^*elu*15/elu15^* (hereafter called *abcc6a*^–/–^) larvae.

**FIGURE 2:**
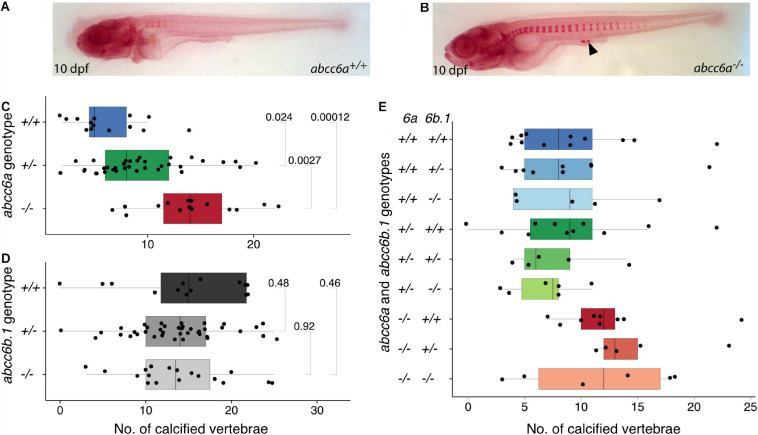
Calcification in single and double mutants of zebrafish *ABCC6* paralogs. **(A,B)** Alizarin Red stained 10 dpf *abcc6a*^+/+^ and *abcc6a*^–/–^ larvae. Ectopic calcification loci in mutants are denoted with arrowhead. **(C)** Quantification of calcified vertebrae in the 10 dpf progeny of *abcc6a*^+/–^ carriers, plotted by genotype. The results show a significant increase in the rate of calcification in the *abcc6* mutants compared to their siblings (*n* = 65, significance levels calculated with Mann-Whitney test). **(D)** Quantification of calcified vertebrae in the 14 dpf progeny of *abcc6b.1^+/–^* carriers, plotted by genotype. Results suggests that impairment of *abcc6b.1* does not affect the rate of vertebral calcification (*n* = 72, significance levels calculated with Mann-Whitney test). **(E)** Quantification of calcified vertebrae in the 14 dpf progeny of *abcc6a^+/–^;abcc6b.1^+/–^* carriers, plotted by genotype (*n* = 68).

At 10 days post fertilization (dpf) the number of calcifying vertebrae was significantly different in *abcc6*^–/–^ mutants stained with Alizarin Red compared to their siblings, and ectopic foci of calcification were also occasionally observed (Figures 2A–C). Ectopic calcification could also be confirmed by an independent, colorimetric method that relies on changes in Ca^2+^ concentration (Supplementary Figures 2C,D). We also tested this phenotype in the offspring of *abcc6a*^–/–^ females, but *MZabcc6a* larvae did not show a more severe phenotype, suggesting that *abcc6a* has no maternal effects (Supplementary Figures 2A,B). Interestingly, we also observed a slight, but significant difference between genotypically wild-type and heterozygote embryos (Figure 2C), similarly to what has been observed in the case of the *grt* mutation (Mackay et al., 2015).

In contrast, *abcc6b.1^*elu*16/elu16^* (hereafter called *abcc6b.1^–/–^*) larvae did not display any differences in the number of calcified vertebrae at 14 dpf (Figure 2D). As the lack of phenotype could be due to putative compensatory effects of *abcc6a*, we created double carrier fish (*abcc6a^+/–^; abcc6b.1^+/–^*) and measured calcification in their progeny. The impaired function of *abcc6a* resulted in increased calcification in every genotype, but we could not identify any effect of *abcc6b.1* on the number of calcified vertebrae at 14 dpf (Figure 2E). (Due to relatively modest sample sizes slight effects would not be apparent here, though.)

We conclude that unlike its paralog *abcc6a*, *abcc6b.1* has modest to no effects on larval calcification.

### Skeletal Defect in *abcc6a* Adult Fish

The effect of Abcc6a impairment on adult skeletogenesis has been described before, but never quantified in detail (Mackay et al., 2015; Van Gils et al., 2018). Also, while the function of Abcc6b.1 does not seem to be required during larval bone formation, it could have an important role during later stages of skeletal development. Therefore we decided to quantify in detail the skeletal development of our single and double mutants using micro-CT.

**FIGURE 3:**
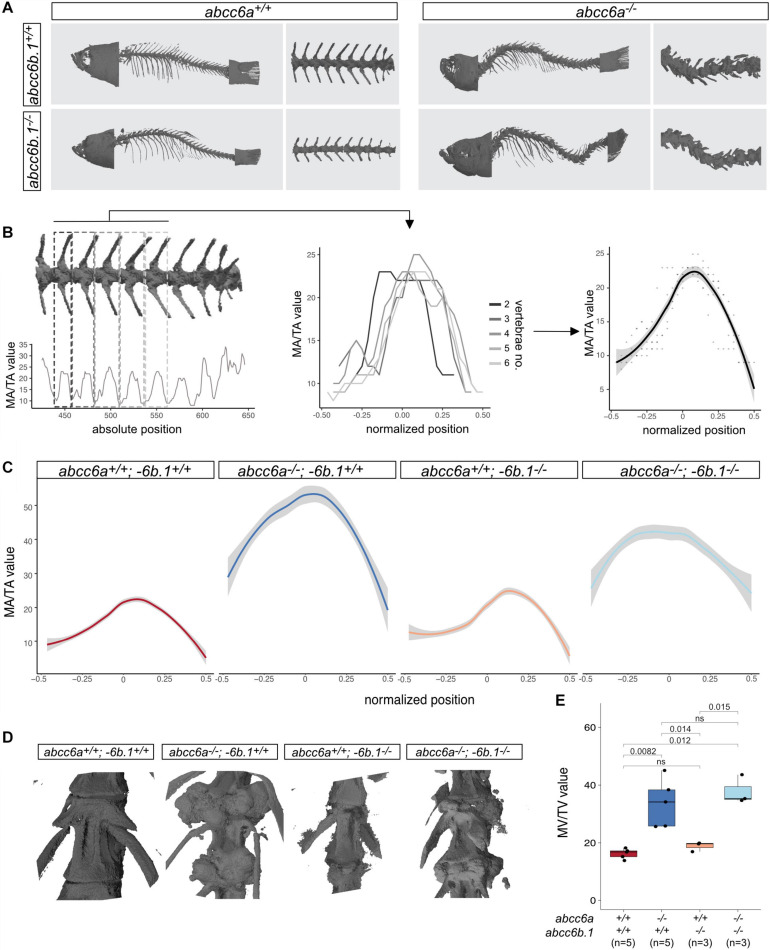
Effects of Abcc6-impairment of skeletal development and life-span. **(A)** Micro-CT pictures of whole fish and partial vertebral column with indicated genotypes. *Abcc6a* mutants (regardless of the *abcc6b.1* genotype) show easily observable, gross skeletal deformities. **(B)** A graphical explanation of the method used to calculate normalized MA/TA plots for each genotype. Briefly, MA/TA values calculated for vertebraes 2–6 were averaged to create the normalized plots. **(C)** Normalized plots of MA/TA values for the indicated genotypes. The results also indicate an increase in the mineralization of the vertebrae of *abcc6a*^–/–^ fish, while mutations in *abcc6b.*1 do not seem to affect this process. **(D)** 3D reconstruction micro-CT images of vertebrae in fish with the indicated genotypes. **(E)** MV/TV measurements for different genotypes. These results also demonstrate the effect of Abcc6a deficiency of the mineralization of skeletal vertebrae. Abcc6b.1 does not appear to be involved in the same process. (Significance levels calculated with *t*-test, ns = not significant).

Scans of whole animals show a striking phenotype in individuals lacking Abcc6a, with a twisted vertebral column and excessive mineralization foci in the ventral side at the attachment points between the individual vertebrae (Figure 3A).

In order to quantify the effect of Abcc6a on mineralization and test if Abcc6b.1 can augment this process first, we measured the relative size of the mineralized area (MA) to the total area (TA) in different mutants. Using the MA/TA values for vertebrae no. 2–6 we created an average MA score for wild-type, single and double mutant adult fish (Figures 3B,C). Our analysis suggests that mutations in *abcc6b.1* does not affect skeletal development, impairment of *abcc6a* caused malformed vertebrae with excessive mineralization (Figure 3C). (Pairwise *t*-tests indicate non-significant differences (*p* = 0.16) between wild-type and *abcc6a*^+/+^; *−6b.1^–/–^* fish, and significant differences (*p* < 2^∗^10^–16^) between these two genotypes and *abcc6a^–/–^;−6b.1^+/+^* and *abcc6a^–/–^;−6b.1^–/–^*, respectively.)

We also calculated the relative mineralized volume (MV) of individual vertebrae in all examined genotypes and similarly to the quantification of MA, we found significant differences between *abcc6a*^+/+^ and *abcc6a*^–/–^ genotypes, regardless of the *abcc6b.1* genotype (Figure 3E).

In the absence of apparent skeletogenic phenotypes, we reasoned that *abcc6b.1* might have undergone neofunctionalization and this new role is independent of regulation of mineralization. Even in this case, however, adverse effects arising from Abcc6b.1 impairment could affect the survival of the animals. To test this we decided to explore if *abcc6b.1* lack-of function has an effect of the survival of the animals. In the progeny of *abcc6b.1^+/–^* parents *abcc6b.1^–/–^* animals were present at Mendelian ratios at 4 months post fertilization, with no apparent phenotype (not shown). Furthermore even in the progeny of double carriers, no significant effect of *abcc6b.1* on survival could be identified within the observed time period (∼400 days) (Supplementary Figure 3).

Based on our observations we conclude that while Abcc6a is involved in the regulation of mineralization, akin to other vertebrate ABCC6 orthologs, Abcc6b.1 might have undergone neofunctionalization and has functions that are not apparent under homeostatic laboratory conditions.

### Significant Mutations in Abcc6b.1

**FIGURE 4:**
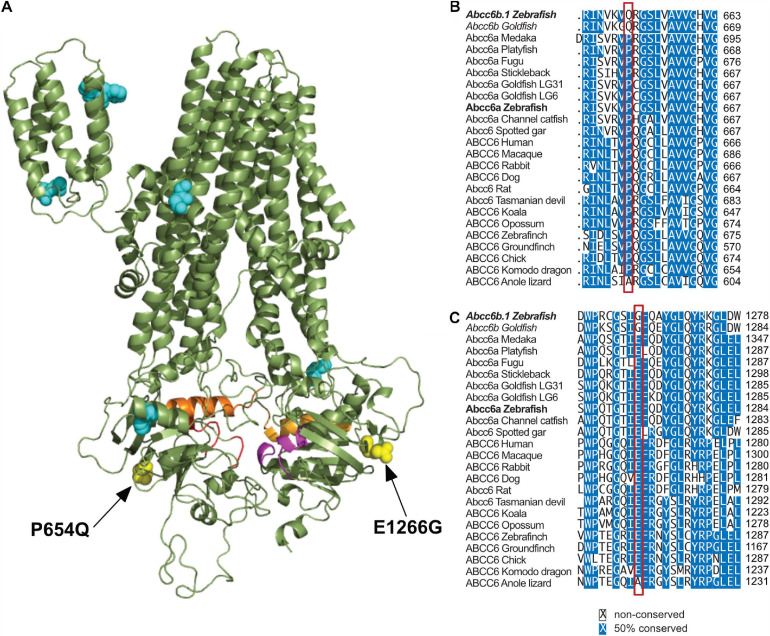
Significant differences in between Abcc6b.1 and canonical ABCC6 sequences. **(A)** Homology model of ABCC6, with conserved residues that are changed in Abcc6b.1 highlighted. **(B)** Alignment of multiple ABCC6 orthologs with the P654Q change highlighted by the red box. **(C)** Alignment of multiple ABCC6 orthologs with the E1266G change highlighted by the red box. These alignments show that P654 and E1266 are almost universally conserved across vertebrates, suggesting that they are important for the function of ABCC6. (Alignments of residues highlighted in cyan in A are detailed in Supplementary Figure 4).

As Abcc6b.1 clearly has non-redundant functions with Abcc6a, we wondered if we could identify mutations that affect residues thought to be important for the transporter function of ABCC6. Using a multispecies comparison approach we were able to identify several residues that are conserved virtually in every other ABCC6/Abcc6a ortholog (including zebrafish Abcc6a) but are divergent in zebrafish and goldfish Abcc6b.1 and Abcc6b, respectively (Figure 4 and Supplementary Figure 4).

Based on *in silico* sequence-structure analysis, two of these mutations, P654Q (equivalent to P669Q in human ABCC6) and E1266G (equivalent to E1290G in human ABCC6) seem especially noteworthy, as they are positioned close to the Walker A motif of NBD1 and NBD2, respectively, and according to our structural analysis they might affect the enzymatic activities of these domains (Figure 4).

## Discussion

In order to create an accurate zebrafish PXE model strain we decided to resolve outstanding issues in the zebrafish genomic assemblies related to the identity and position of possible *ABCC6* orthologs and test if the functional paralogs have redundant function in mineralization.

We have been able to reconstruct the poorly assembled region of chromosome 6 where *abcc6b.1* and *abcc6b.2p* are situated and showed that of the two genes only the former is functional, and the latter is a pseudogene (Figures 1A–D). This pattern not observed in the genome of the related catfish suggests that relatively recently a segmental duplication occurred at this position of the zebrafish genome and one of the copies degenerated later (Figure 1E). Similarly, the human *ABCC6* is abutted by two pseudogenes that originated through segmental duplications (Pulkkinen et al., 2001); however, they are supposed to be results of more recent, unrelated genomic events (Symmons et al., 2008).

Intriguingly, while the presence of *abcc6a* and the original *abcc6b* would be parsimonious with their origin after the TGD, our results suggest that this might not be necessarily the case.

As interspecies genomic alignments show that multiple teleost species have only a single syntenic *abcc6* region in their genomes (Figures 1E,E’), it is possible that one half-genome with the second *abcc6* genomic cluster was lost shortly after the TGD. If this is indeed the case, than the evolution of the current zebrafish *abcc6* complement involved two segmental duplication events, with the first being followed by a chromosomal translocation with a break point between the duplicates. This rearrangement could have occurred somewhere at origins of the Ostariophysan superorder, in the common ancestor of channel catfish and zebrafish. This is why both of these species carry a split form of the ancestral *abcc6* chromosomal locus (Figures 1E,E’).

However, the presence of a second *abcc6* cluster in the genome of the Atlantic herring also suggests an alternative hypothesis. While this syntenic region might be the result of a lineage-specific chromosomal duplication event, it is also possible that post-TGD differential gene-loss followed different trajectories in the ancestors of the Otocephala and Euteleostei clades. Unlike all other observed species, zebrafish, channel catfish and the Atlantic herring all belong to the former clade, and their common ancestors might have preserved both half-genome fragments with *abcc6*, whereas Euteleostei have lost one. Later the ancestral ostariophysan lost the downstream members of the *abcc6a* locus and the upstream members of the *abcc6b* locus, respectively. Such a sequence of events would also be parsimonious with the observed pattern.

As further teleost genomes will become available, we will be able to discern between these two hypotheses.

Using a CRISPR/Cas9-based methodology we were able to create frameshift mutations in both functional zebrafish *ABCC6* paralogs, *abcc6a*, and *abcc6b.1*. The phenotypic analysis of single and double mutants, both at larval and adult stages suggested that whereas *abcc6a* has major roles in mineralization, as has been suggested by previous studies (Mackay et al., 2015; Van Gils et al., 2018; Sun et al., 2021), *abcc6b.1* appears to be dispensable for this process under normal conditions. In the absence of Abcc6a function we observe precocious mineralization as early as 10 dpf (Figure 2), and in adults this process leads to deformed vertebrae and ectopic mineralization foci deposited in between the vertebrae leading to a characteristic phenotype (Figure 3), reminiscent to some scoliosis models (Grimes et al., 2016).

Our results also suggest that earlier morphant phenotypes ascribed to *abcc6a* loss-of-function (Li et al., 2010) could be due to off-target effects of the morpholinos. Morpholino treatments are known to be prone to such effects, which result in non-specific phenotypes (Schulte-Merker and Stainier, 2014; Kok et al., 2015). The discovery of genetic compensation in multiple mutant lines, however, also demonstrated that lack of phenotypes in particular mutants could be also due to the upregulation of paralogous genes by transcriptional adaptation (Rossi et al., 2015; El-Brolosy et al., 2019; Sztal and Stainier, 2020). Here, we show that no upregulation of *abcc6a* can be detected in *abcc6b.1^–/–^* background thus the absence of phenotype cannot be explained with transcriptional adaptation. Furthermore, we could not detect the “*abcc6a* morphant” (or indeed any) embryonic phenotype in either the *MZabcc6a* mutants or in our double mutant embryos, also suggesting that *abcc6a* and *abcc6b.1* are unlikely to have functions during early development.

Of note, while we could not detect a function for Abcc6b.1 during homeostatic conditions, neither in mineralization, nor in lifespan (Supplementary Figure 3), it is possible that it has yet uncovered roles in the stress response. Indeed, recent results show an upregulation of *abcc6b.1* in a zebrafish chordoma model (D’Agati et al., 2019).

Using *in silico* approaches we have also identified some key residues that have been changed in the Abcc6b.1 sequence compared to the archetypical ABCC6. Two of these changes, P654Q and E1266G appear in key positions of the NBD1 and NBD2 and they might critically alter the function of the transporter. It has to be noted, however, no human mutations are known to be related to these two residues (equivalent to P669 and E1290 in human ABCC6), so later empirical studies have to clarify the consequences of these two changes in the sequence and provide unequivocal evidence about the role of Abcc6b.1 in zebrafish physiology.

Over the past decade several highly influential studies have demonstrated the usefulness of the zebrafish model in the preclinical phases of drug development and documented the advantages this versatile model in the framework of the 3Rs (replacement, reduction, refinement) (for comprehensive reviews on this subject see Cassar et al., 2020 and Macrae and Peterson, 2015). External fertilization and fast development means that only one or 2 weeks after fertilization we can easily observe the well developed internal organs of zebrafish larvae and also assay their behavior. The fecundity of zebrafish females also makes this species ideal for high-throughput experimental approaches (Stewart et al., 2015; Cassar et al., 2020). The mutant lines that we have created can be used not only to probe the function of the zebrafish ABCC6 paralogs, but are also ideal to test small molecular drugs that could ameliorate the PXE phenotype as other studies have shown (Mackay et al., 2015). Etidronate and PPi are obvious choices for such tests (Dedinszki et al., 2017; Kranenburg et al., 2018; Li et al., 2018), but high-throughput screening of compounds approved by the Federal Drug Administration (FDA) could help to find putative drugs that can be quickly repurposed to help PXE patients as happened recently for several other diseases (Cully, 2019). The existence of well defined larval phenotypes in the *abcc6a* mutant zebrafish will also significantly shorten the timeframe to test particular drugs from the 4–5 months, typical in mice, to ∼2 weeks.

Overall the models we have developed offer a cost-effective approach for the discovery and/or repurposing of drugs and will significantly shorten the timeframe for the development of novel PXE therapies.

## Materials and Methods

### Fish Husbandry

Wildtype and mutant fish lines were maintained in the animal facility of ELTE Eötvös Loránd University according to standard protocols (Westerfield, 2000; Aleström et al., 2019). All protocols used in this study were approved by the Hungarian National Food Chain Safety Office (Permit Number: PE/EA/2026-7/2017).

### Genome Editing and Genotyping

To induce indel mutations in the *abcc6a* and *abcc6b.1* genes we used the CRISPR/Cas9 system as described (Gagnon et al., 2014). The targeted site within the 2nd exon of *abcc6a* and the 6th exon of *abcc6b.1*, respectively (with the PAM sequence in bold): GGCAGCCGACCTCGGCCATGG and AGAAGCATCCTCAACTGGACATGG (Supplementary Figure 1).

Adult and larval zebrafish were genotyped using the HotShot method (Meeker et al., 2007). The genotyping of the *abcc6a* wild-type and mutant (*elu15*) alleles was achieved using the 5′-CC ATCTCTACTGCCATGGCC-3′ and 5′-CATCTCTACTGCCAT GGGTC-3′ forward primers, respectively, in combination with the 5′-CTGAGGGGTCGAGTTCAAACTT-3′ reverse primer. For Sanger sequencing the latter reverse primer was used in combination with the 5′-CCATCTCTACTGCCATGGCC-3′ forward primer.

The genotyping of the *abcc6b.1* wild-type allele was achieved using the 5′-ACAACCTGTCAGCGTCTTTGTC-3′ forward and the allele-specific 5′-AGAAGCATCCTCAACTGGACA-3′ reverse primers. Genotyping of the *elu16* allele was performed with the allele-specific 5′-TCCACTAGAACCCCTAGCAC-3′ forward primer in combination with the 5′-AACAA TGGGCCAAACTGCAACA-3′ reverse primer. For Sanger sequencing the critical region was amplified with the two primers that are not allele-specific.

### PacBio Sequencing and Data Analysis

A gynogenetic diploid, double heterozygous adult was generated by heat shock according to previously established protocols (Westerfield, 2000). The genomic DNA was isolated using the Qiagen MagAttract HMW DNA kit (Qiagen cat#: 67563). Small molecular weight DNA was removed with BluePippin size selection (Sage Science cat#: HEX0004). A genomic DNA library was prepared and sequenced to 100× genomic coverage (1,500 gb) using the PacBio Sequal II system. Whole genome assembly was performed using the Canu assembly software (Koren et al., 2017), and the contiguous regions of interest was pulled out based on homology to the *abcc6a* or *abcc6b* genes. Raw sequence data has been deposited to the NCBI SRA public database under BioProject identifier PRJNA698636 and will be made public upon the acceptance of the manuscript. Reviewer link: https://dataview.ncbi.nlm.nih.gov/object/PRJNA698636?reviewer=m1tk397880d159so2e1c248u77. The sequence of the reconstructed genomic region, with annotated *abcc6b.1* and *abcc6b.2p* has been deposited to GenBank (submission ID: 2425245).

### *In silico* Structure Analysis

A homology model of the human ABCC6 protein was created as described earlier (Kozák et al., 2020). Briefly, one hundred structures were generated by Modeler v9.24 (Webb and Sali, 2016) using four high resolution structures of bovin ABCC1 (MRP1) as template. Membrane orientation of best models were determined by the TMDET algorithm (Tusnády et al., 2005) and CHARMM-GUI (Jo et al., 2008) was applied to generate lipid environment around the protein. For energy minimization Gromacs version 2018.8 (Hess et al., 2008) was utilized. The quality of the final model were checked by PROCHECK-NMR (Laskowski et al., 1996).

### Alizarin Red Stainings

Larvae were fixed for 2 days at 4°C in 4% paraformaldehyde (PFA) dissolved in phosphate-buffered saline (PBS) solution. Samples were washed twice in ddH_2_O and incubated for 30 min in a mixture of 60 μl 30% H_2_O_2_ and 1.94 ml “Solution 2” (1% KOH, 2% Triton X-100) to remove pigmentation. Repeated washing in ddH_2_O was followed by an overnight incubation at 4°C in 1:1 mixture of Alizarin Red solution and “Solution 2.” The next day excess staining solution was discarded and the samples were washed in “Destain solution” (20% glycerol, 0.25% KOH). For long term storage larvae were moved into 50% glycerol, 0.25% KOH.

### Lifespan Analysis

Fish (*n* = 148) were genotyped at 3 month age and raised in separate tanks, according to each of the nine genotypes. At ∼400 days age all surviving fish were culled and fixed in 4% paraformaldehyde (PFA) in PBS for later micro-CT measurements. Each fish was re-genotyped at this stage by a combination of allele-specific PCR and Sanger sequencing, when deemed necessary.

### Micro-CT Analysis

Adult zebrafish were anesthetized, culled according to standard protocols. Each individual was genotyped by fin-clipping and fixed and stored in 4% paraformaldehyde (PFA) dissolved in phosphate-buffered saline (PBS) at 4°C. Micro-CT analysis (Skyscan 1272, Bruker, Kontich, Belgium) was performed as described before (Csete et al., 2019). Whole-body scans were acquired using a 100 kV and 100 μA X-ray source without filter followed by reconstruction with the SkyScan NRecon software (Bruker, Kontich, Belgium), resulting in a 5 μm or 20 μm voxel size. Further analysis was performed using the Skyscan CTAn (Bruker, Kontich, Belgium). The lower threshold of binary images was set to an absolute value of 95 or 105. Quantitative analysis was performed on the precaudal vertebraes of the fish using an axial cylinder of a diameter of 1,000 μm around the center of vertebraes. Determined parameters were percent mineralized volume (MV/TV) in the total volume of interest and percent mineralized area (MA/TA) in every section.

### Multiple Sequence Alignments, Data Analysis and Visualization

Statistical analysis, multiple sequence alignments and visualization was performed in R (R Core Team, 2018) using the *msa, fishtree* and *ggplot2* packages (Bodenhofer et al., 2015; Wickham, 2016; Chang et al., 2019) and the online Genomicus database (Louis et al., 2013). To display phylogenetic relationships we used the *jsPhyloSVG* script (Smits and Ouverney, 2010). All figures have been assembled in Affinity Designer (Serif Europe).

## Data Availability Statement

The datasets presented in this study can be found in online repositories. The names of the repository/repositories and accession number(s) can be found below: NCBI BioProject, accession no: PRJNA698636.

## Ethics Statement

The animal study was reviewed and approved by the Hungarian National Food Chain Safety Office.

## Author Contributions

MV, AV, and AM: conceptualization. MV, DCs, GT, ZC, and SB: data curation. AV, MV, AM, SB, and GT: funding acquisition. MV, DCz, KP, ZG, VL, HG, DCs, KF, ZC, SB, and GT: investigation. MV, DCs, GT, SB: methodology. MV, AM, AV, and SB: project administration. MV and AM: supervision. MV, AV, and KF: writing – original draft. All authors contributed to the article and approved the submitted version.

## Conflict of Interest

The authors declare that the research was conducted in the absence of any commercial or financial relationships that could be construed as a potential conflict of interest.
