# Supplementary material for: A New Zebrafish Model for Pseudoxanthoma Elasticum
Source: Front Cell Dev Biol. 2021 Mar 9;9:628699. doi: 10.3389/fcell.2021.628699 (PMC7985086; doi:10.3389/fcell.2021.628699)
Supplement: Supplementary file 1 [file Table_1.DOCX]

Supplementary Material

# Supplementary Materials and Methods

## Colorimetry

For colorimetric detection assay the Stanbio Calcium (CPC) Liquicolor Test (Stanbio Laboratory, cat no. 0150-250) was used, according to the manufacturer’s protocols. Adult and larval tissues were homogenized in 0.15N HCl at room temperature. A total of 354 mg wild-type and 320 mg mutant muscle tissue was used for the measurements. Normalized colorimetry values were calculated by homogenizing a total of 3 or 5 larvae of a given genotype and dividing the result with the number of pooled individuals.

# Supplementary Figures


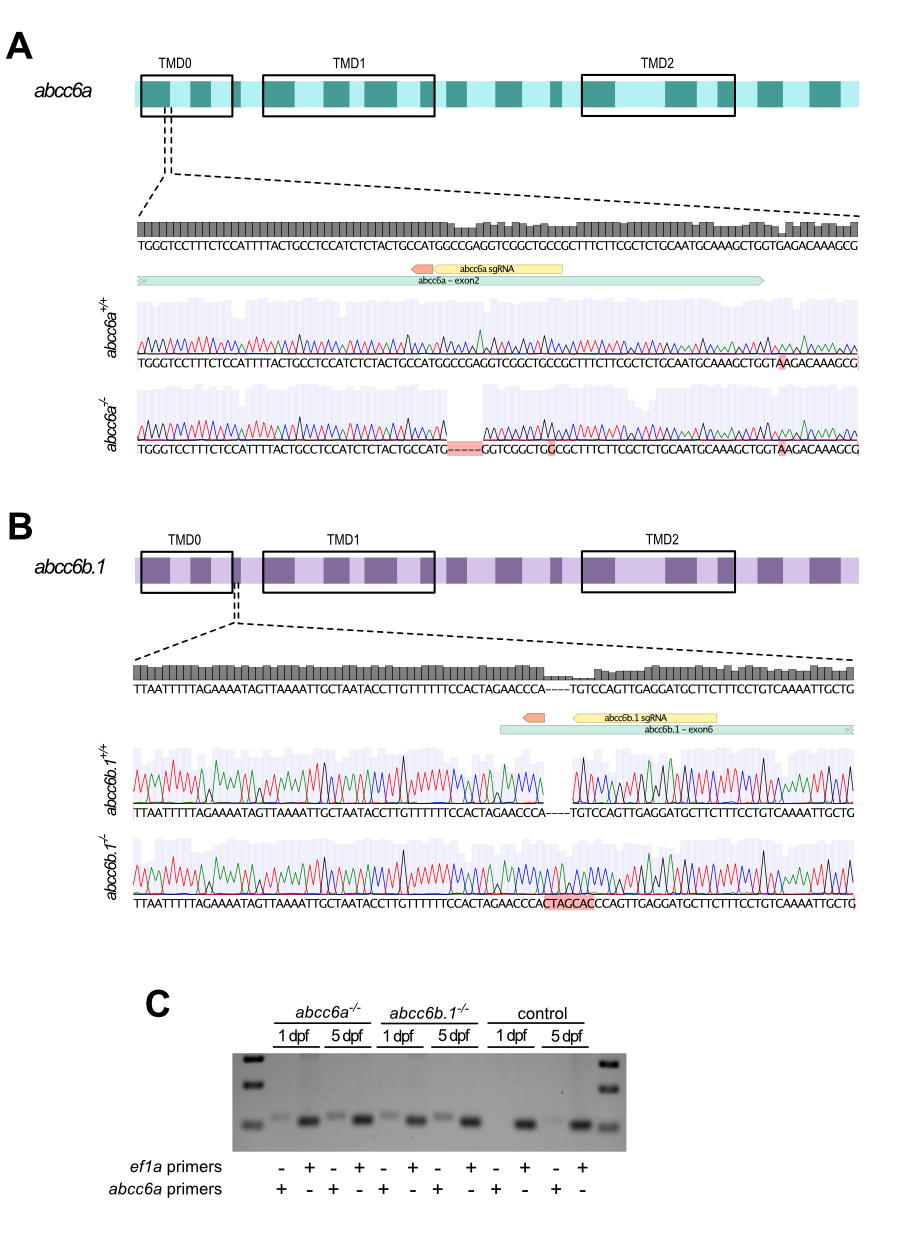


**Supplementary Figure 1: Generation of mutant alleles.** (A) The position of the c.175_179delGCCGA (*elu15*) mutation in the *abcc6a* gene and typical sequenograms of wild-type and homozygous mutant animals. (B) The position of the c.616_618delTGTinsCTAGCAC (*elu16*) mutation in the *abcc6b.1* gene and typical sequenograms of wild-type and homozygous mutant animals. (C) RT-PCR amplification of *abcc6a* and *ef1a* in 1 dpf and 5 dpf *abcc6a^-/-^* and *abcc6b.1^-/-^* samples.


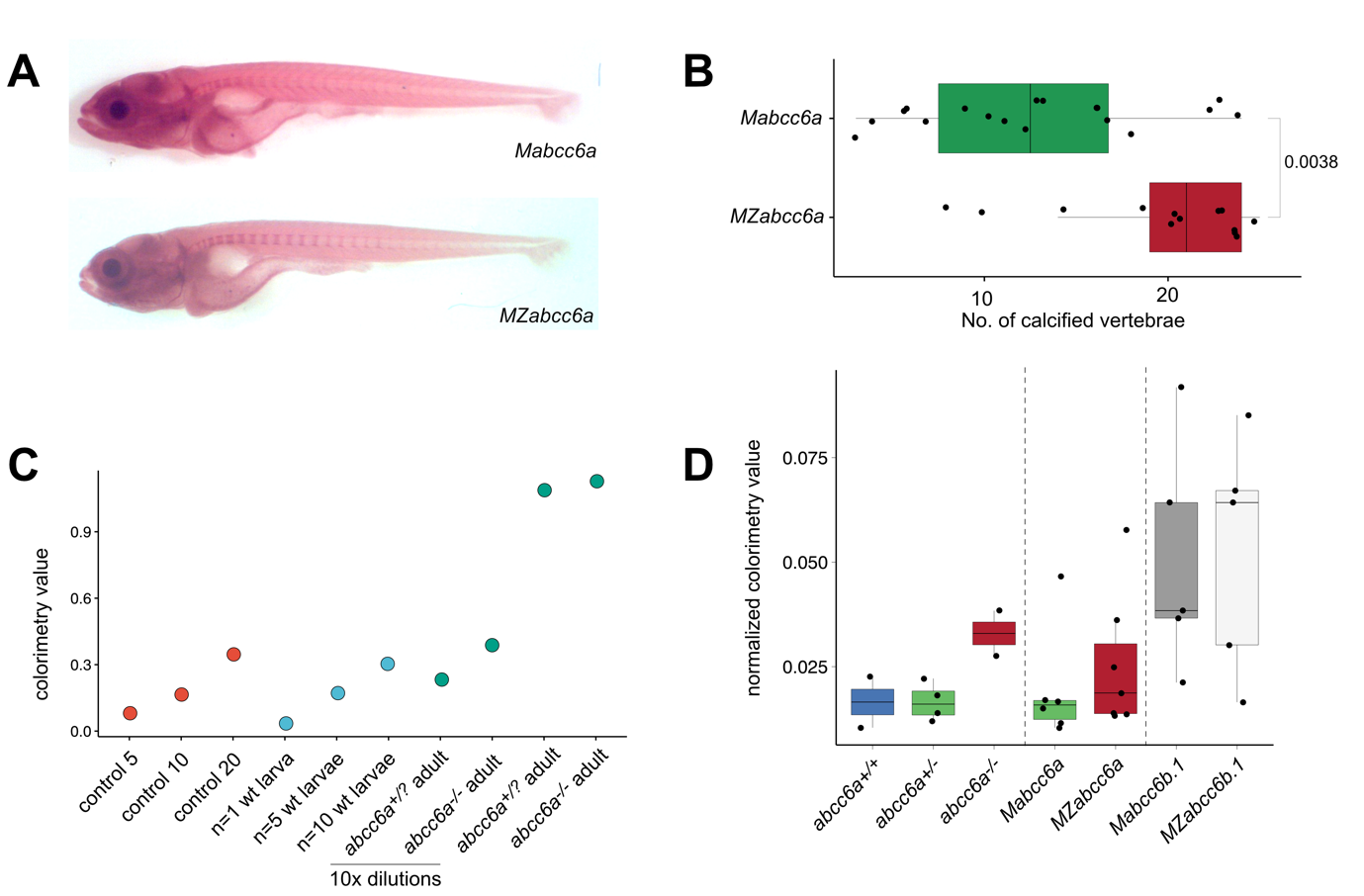


**Supplementary Figure 2: Analysis of *MZabcc6a* larvae and colorimetric measurements.** (A) Alizarin Red staining of 14 dpf *Mabcc6a* and *MZabcc6a* larvae. (B) Quantification of calcified vertebrae in *Mabcc6a* (n=17) and *MZabcc6a* (n=13) animals. (Statistical significance was calculated with Mann-Whitney test.) (C) Colorimetric detection essay in larval and adult tissues of the indicated genotype. (D) Colorimetric detection of mineralization in larval tissues of the indicated genotypes.


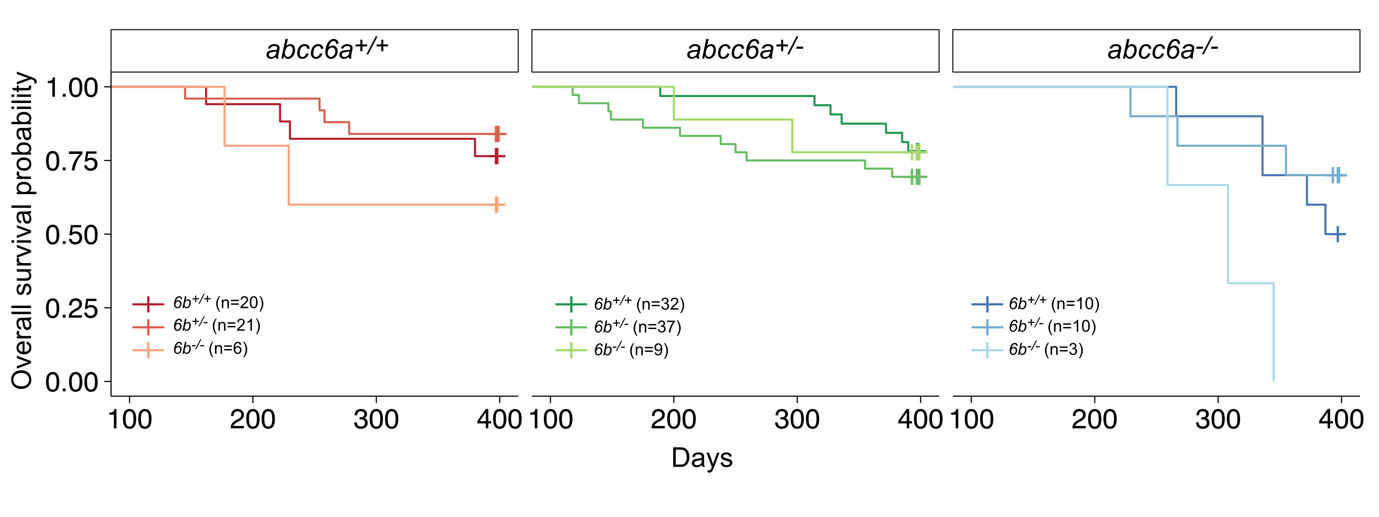


**Supplementary Figure 3: Lifespan analysis of different combinatorial genotypes.** No significant differences could be detected in the survival of *abcc6b.1^-/-^* fish, regardless of the *abcc6a* genotype.


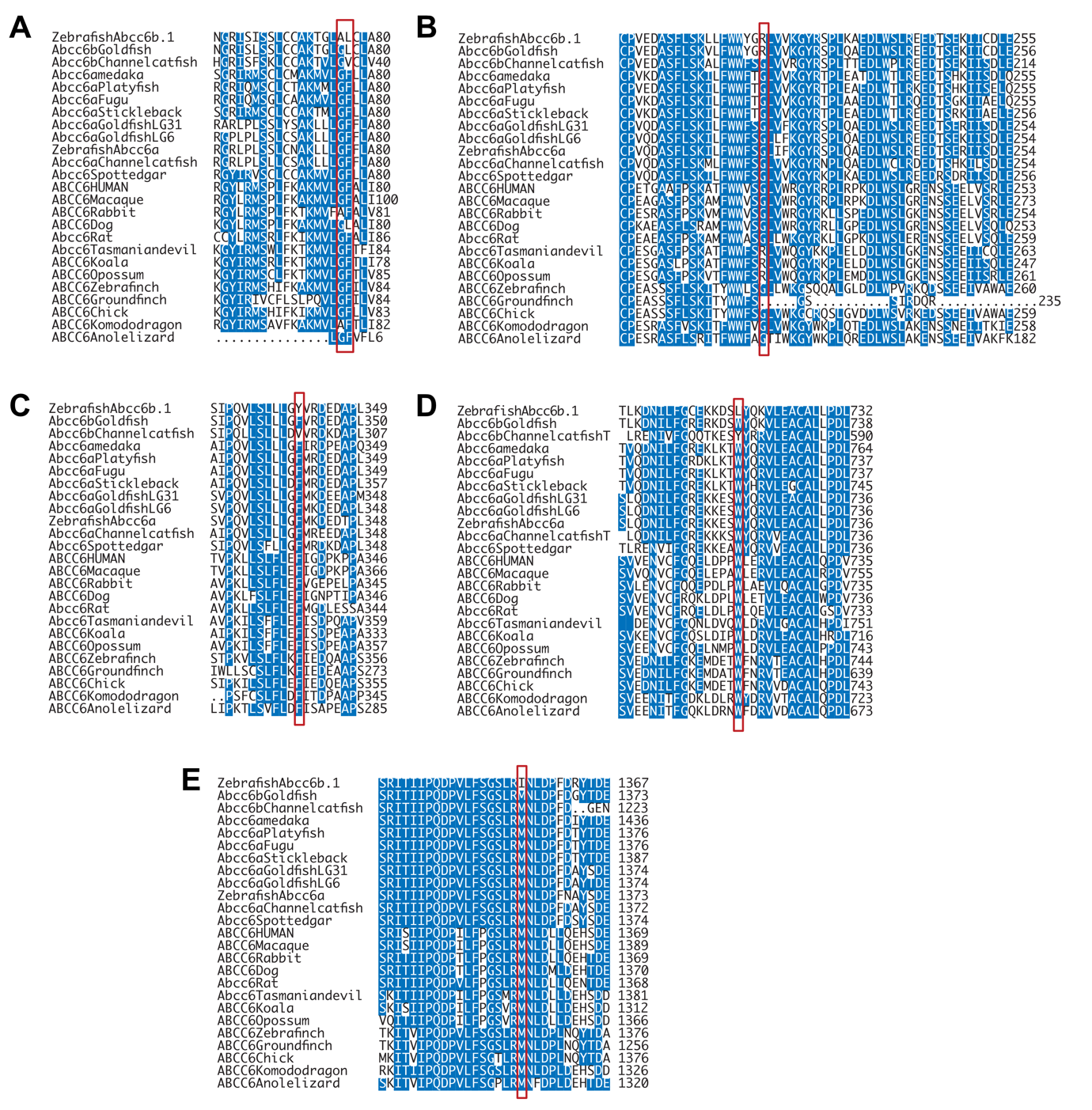


**Supplementary Figure 4: Further “canonical” ABCC6 residues changed in Abcc6b.1.** (A) Alignment of multiple ABCC6 orthologs with the G76A and F77L mutations (equivalent to human G76A and F77L, respectively) highlighted with the red box. (B) Alignment of multiple ABCC6 orthologs with the G223R mutation (equivalent to human G221R) highlighted with the red box. (C) Alignment of multiple ABCC6 orthologs with the F341Y mutation (equivalent to human F338Y) highlighted with the red box. (D) Alignment of multiple ABCC6 orthologs with the W718Y mutation (equivalent to human W721Y) highlighted with the red box. (E) Alignment of multiple ABCC6 orthologs with the M1356I mutation (equivalent to human M1358I) highlighted with the red box.
